# Adrenal Insufficiency due to Disseminated *Cryptococcus* in an Immunocompetent Individual

**DOI:** 10.1155/crie/8534408

**Published:** 2026-01-21

**Authors:** Jeremy A. Knott, Zoran Apostoloski

**Affiliations:** ^1^ Department of Endocrinology, Wollongong Hospital, Sydney, NSW, Australia, nsw.gov.au; ^2^ Faculty of Medicine, The University of New South Wales Sydney, Sydney, NSW, Australia, unsw.edu.au

**Keywords:** adrenal insufficiency, disseminated *Cryptococcus*, immunocompetent

## Abstract

Primary adrenal insufficiency due to infiltrative fungal infections, such as *Cryptococcus neoformans*, is rare, particularly in immunocompetent patients. We present a case of an immunocompetent 61‐year‐old man who presented with bilateral adrenal enlargement and adrenal insufficiency, with a 3‐month history of generalised fatigue, weight loss and dizziness, as well as initial hyponatraemia and hyperkalaemia. Adrenal computed tomography (CT) revealed bilaterally enlarged adrenals, and fluorodeoxyglucose positron emission tomography (FDG‐PET) revealed increased peripheral metabolic surrounded by a photopenic core suggestive of central necrosis. Adrenal biopsies were consistent with *Cryptococcus*. Serum cryptococcal antigen (CrAg) testing was strongly positive (titres 1:1280) as was cerebrospinal fluid analysis (titre 1:160). The patient was commenced on hydrocortisone and fludrocortisone, with improvement in symptoms. Treatment involved induction therapy with intravenous liposomal amphotericin‐B 4 mg/kg daily and 5‐flucytosine 25 mg/kg four times daily for 2 weeks, followed by consolidation therapy with fluconazole. This case highlights the importance of considering disseminated cryptococcosis in immunocompetent individuals presenting with adrenal insufficiency. Early diagnosis and appropriate antifungal therapy are crucial.

## 1. Introduction

Primary adrenal insufficiency is a potentially life‐threatening condition with the most common cause attributed to autoimmune adrenalitis in developed countries [1]. Differentiating the underlying cause of adrenal insufficiency can be challenging due to the nonspecific clinical presentation and broad range of aetiologies. Infiltrative fungal infections, such as *Cryptococcus neoformans*, causing adrenal insufficiency due to direct adrenal involvement are exceedingly rare, particularly in immunocompetent patients. This case outlines an immunocompetent 61‐year‐old man who presented with adrenal insufficiency and persistent bilateral adrenal enlargement due to disseminated *Cryptococcus* infection.

## 2. Case Report

A 61‐year‐old Caucasian male presented with a 3‐month history of generalised weakness, fatigue, weight loss and postural dizziness. There were no infective symptoms. He had no significant background medical history and was not on any medications. On physical examination, he appeared fatigued and was afebrile, hypotensive (blood pressure 92/73 mmHg) and had dry oral mucosa. His body mass index was 17 kg/m^2^.

**Figure 1:**
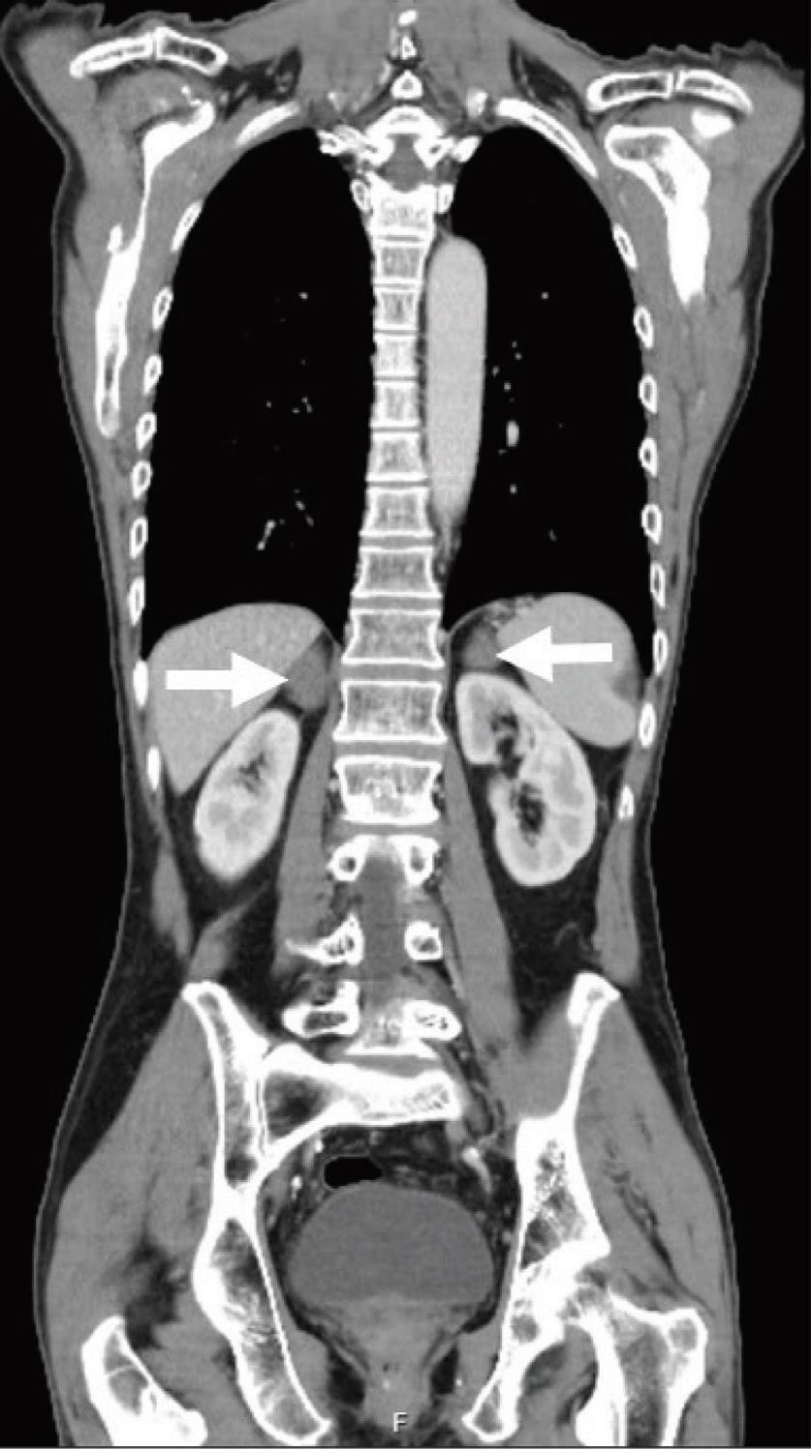
An abdominal computed tomography (CT) scan revealed bilateral adrenal enlargement (arrows) of unclear cause on initial imaging.

Preliminary serology revealedhyponatraemia (Na 123 mmol/L; reference interval [RI],135–145 mmol/L), hyperkalaemia (K 5.5 mmol/L; RI, 3.5–5.2 mmol/L) and low morning cortisol (205 nmol/L; RI, 166–507 nmol/L), with elevated ACTH (216.2 ng/L; RI, 7.2–63.3 ng/L) confirming primary adrenal insufficiency. Initial abdominal computed tomography (CT) demonstrated bilateral adrenal enlargement of unclear cause (Figure 1). Steroid replacement with hydrocortisone (20 mg morning and 10 mg midday) and fludrocortisone (100 mcg daily) led to resolution of symptoms and biochemical correction.

Repeat imaging as an outpatient showed persistent adrenal enlargement of unclear cause. A further fluorodeoxyglucose positron emission tomography (FDG‐PET)/CT scan revealed increased FDG uptake (SUVmax 5.1 on the right, 3.3 on the left), with increased peripheral metabolic uptake surrounded by a relatively photopenic core region suggestive of central necrosis (Figure 2). Phaeochromocytoma was excluded prior to biopsy with normal plasma metanephrines (<50 pmol/L; RI <447 pmol/L), normetanephrines (152 pmol/L; RI <1010 pmol/L) and 3‐methoxytyramine (<25 pmol/L; RI <100 pmol/L). CT‐guided adrenal biopsies revealed *Cryptococcus* organisms (Figure 3), and *Cryptococcus neoformans* DNA was detected by PCR. Serum cryptococcal antigen (CrAg) testing was strongly positive (titres 1:1280) as was cerebrospinal fluid analysis (titre 1:160), indicating disseminated infection.

Figure 2A FDG‐PET scan revealed avidity in the right (SUVmax 5.1) and left (SUVmax 3.3) adrenal glands (arrows) with increased peripheral metabolic uptake surrounded by a relatively photopenic core region suggestive of central necrosis. (A) Coronal maximum‐intensity projection image. (B) Axial PET/CT fusion image.

**Figure:**
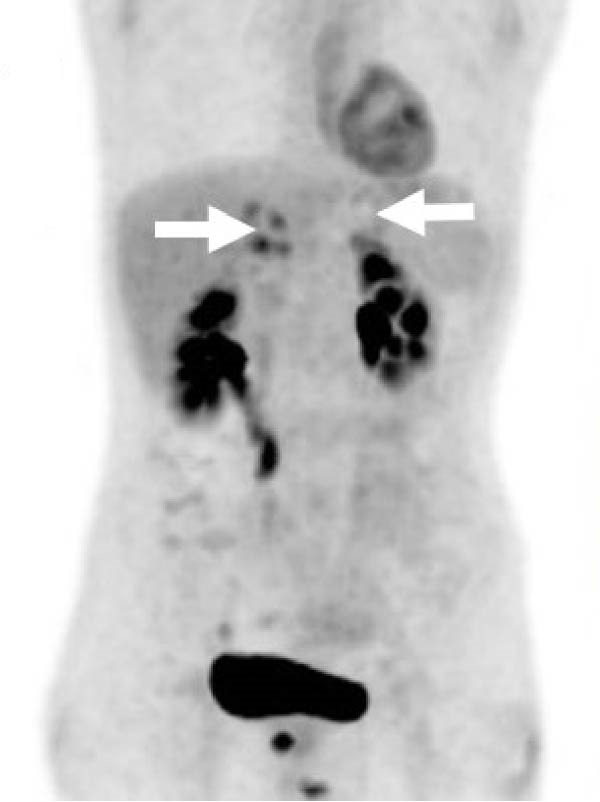
(A)

**Figure:**
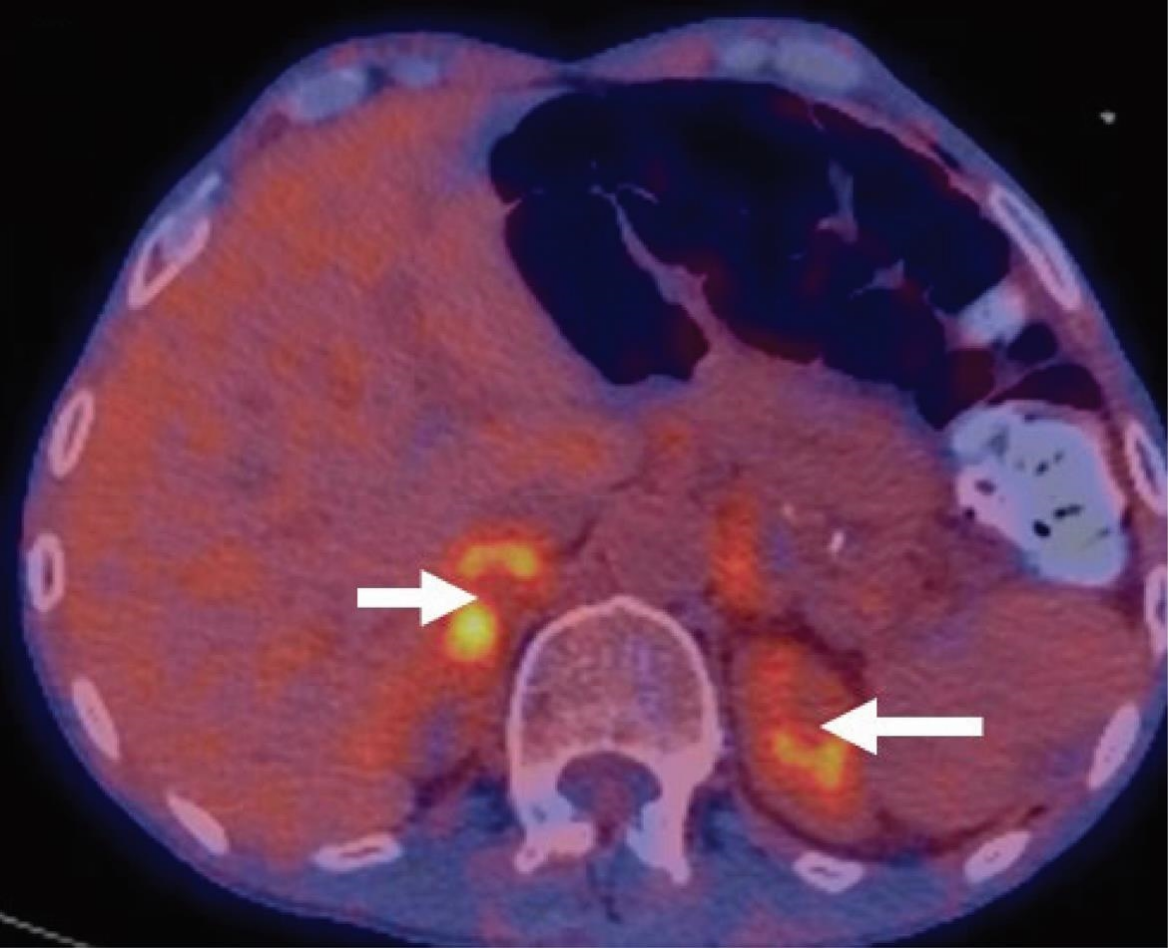
(B)

Figure 3Diagnostic adrenal biopsies revealed *Cryptococcus* organisms present on Grocott methenamine silver stain (A). The *Cryptococcus* polysaccharide capsule can be visualised on mucicarmine stain (B). Haematoxylin and eosin stain (C) revealed areas of diffusely necrotic tissue.

**Figure:**
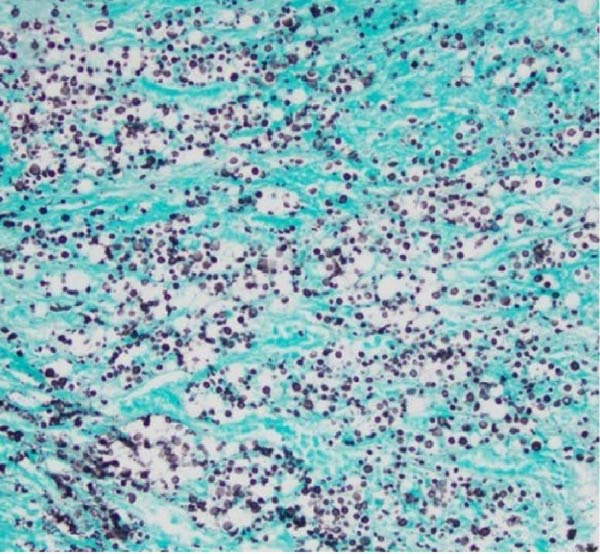
(A)

**Figure:**
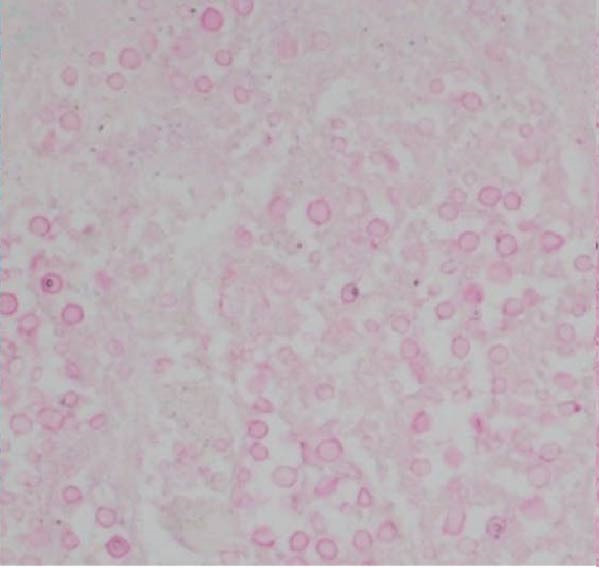
(B)

**Figure:**
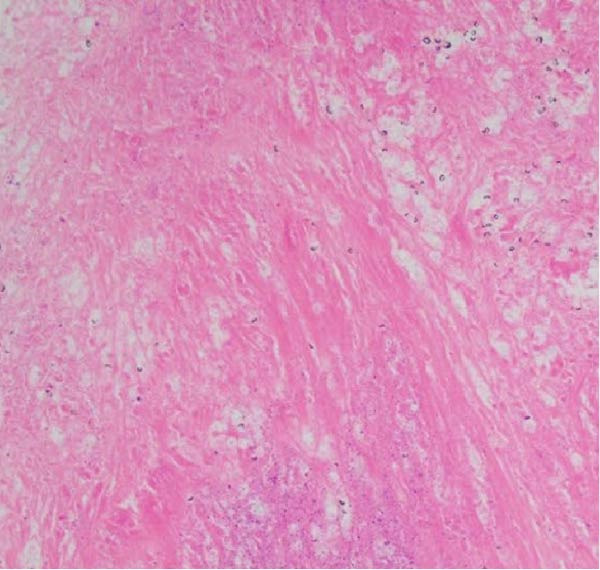
(C)

The patient had normal absolute neutrophil (3.63 × 10^9^/L; RI, 1.7–7.0 × 10^9^/L) and total lymphocyte counts (2.52 × 10^9^/L; RI, 1.5–4.0 × 10^9^/L), with no evidence of chronic illness such as diabetes mellitus (HbA1c 5.4%; RI, 4%–6%) to suggest secondary immunosuppression. HIV antigen/antibody serology and an interferon‐gamma release assay for *Mycobacterium tuberculosis* were negative. Further immunological evaluation performed by the infectious diseases team demonstrated normal T‐cell subsets, including a preserved CD4^+^ count (0.93 × 10^9^/L; RI, 0.7–1.2 × 10^9^/L) and CD4/CD8 ratio (4.5), which was not suggestive of a primary T‐cell defect. Tests for anti‐granulocyte–macrophage colony‐stimulating factor antibodies and anti‐interferon gamma autoantibodies, which have been associated with increased susceptibility to cryptococcal infection, were negative.

Treatment involved induction therapy with intravenous liposomal amphotericin‐B 4 mg/kg daily and 5‐flucytosine 25 mg/kg four times daily for 2 weeks, followed by consolidation and maintenance therapy with fluconazole for 12–18 months. At the time of writing, the patient remains on maintenance hydrocortisone (20 mg morningand 10 mg midday) and fludrocortisone 100 mcg morning, with resolution of symptoms of their adrenal insufficiency.

## 3. Discussion

Disseminated *Cryptococcus* with adrenal involvement causing primary adrenal insufficiency is rare, particularly in immunocompetent individuals. *Cryptococcus neoformans* typically occurs in immunocompromised hosts as meningoencephalitis or lung disease [1]. In Australia, the leading cause of primary adrenal insufficiency is autoimmune adrenalitis, but infectious, infiltrative and metastatic causes may also be considered, particularly in the presence of bilateral adrenal enlargement [2].

The role of functional imaging in differentiating a potential infiltrative cause of adrenal insufficiency is not yet established. The destructive infiltrative process from *Cryptococcus* may reveal a distinct pattern on FDG‐PET/CT imaging with significant metabolic uptake along the adrenal peripheries with areas of central necrosis [3], as seen in our case. FDG‐PET/CT may also be useful to assess disease distribution and in guiding biopsy. This pattern may also help differentiate from malignancy or lymphoma, which often present with more homogeneous or diffuse uptake [3, 4].

Histopathology remains the gold standard for diagnosing adrenal cryptococcosis, but non‐invasive diagnostics play a pivotal role in raising clinical suspicion. CrAg testing is highly sensitive and specific, particularly in disseminated disease, and should be routinely considered in patients with bilateral adrenal enlargement and biochemical adrenal insufficiency of unclear cause [2, 5]. In our case, CrAg testing from serum was strongly positive (titre 1:1280), and earlier recognition for CrAg testing may expedite diagnosis, reduce reliance on invasive procedures and enable prompt initiation of antifungal therapy, potentially improving outcomes.

Despite antifungal clearance, recovery of adrenal function is often unlikely in infiltrative fungal infections due to irreversible destruction of adrenal tissue. In patients with bilateral adrenal disease, it is not always clear if antifungal cryptococcal therapy will result in recovery of adrenal function. In cases where primary adrenal insufficiency is secondary to a destructive disease process, adrenal recovery is unusual [5].

This case underscores the importance of considering fungal infections in the differential diagnosis of bilateral adrenal disease—even in immunocompetent patients. Functional imaging, tissue biopsy and targeted antigen testing are complementary tools that guide accurate and timely diagnosis. Early recognition and a suggested low threshold for CrAg testing, are critical to avoid delays in initiating antifungal therapy.

### 3.1. Learning Points

1. Bilateral adrenal enlargement in the context of primary adrenal insufficiency should prompt consideration of atypical infections such as *Cryptococcus neoformans*, even in immunocompetent patients.
2. Functional imaging (FDG‐PET/CT) may aid in distinguishing infiltrative infectious processes from malignancy and can help guide biopsy.
3. In cases of cryptococcal adrenalitis, adrenal function rarely recovers despite antifungal treatment, and long‐term steroid replacement is typically required.

## Author Contributions

**Jeremy A. Knott**: writing – original draft. **Zoran Apostoloski**: patient diagnosis, treatment, management.

## Funding

No funding was received for this manuscript.

## Disclosure

Our case report was presented as a conference poster presentation at the Endocrine Society Australia in 2024, which can be viewed here: https://esa-srb-anzbms-2024.p.asnevents.com.au/days/2024-11-11/abstract/107311 [6].

## Consent

Written informed consent was obtained from the patient to publish this report in accordance with the journal’s patient consent policy.

## Conflicts of Interest

The authors declare no conflicts of interest.

## Data Availability

The data that support the findings of this study are available from the corresponding author upon reasonable request.
